# Rationale and design of the Innsbruck Diabetic Kidney Disease Cohort (IDKDC)—a prospective study investigating etiology and progression of early-stage chronic kidney disease in type 2 diabetes

**DOI:** 10.1093/ckj/sfae109

**Published:** 2024-04-11

**Authors:** Clemens Plattner, Sebastian Sallaberger, Jan-Paul Bohn, Claudia Zavadil, Felix Keller, Afschin Soleiman, Martin Tiefenthaler, Gert Mayer, Markus Pirklbauer

**Affiliations:** Department of Internal Medicine IV – Nephrology and Hypertension, Medical University of Innsbruck, Innsbruck, Austria; Department of Internal Medicine IV – Nephrology and Hypertension, Medical University of Innsbruck, Innsbruck, Austria; Department of Internal Medicine V – Haematology and Oncology, Medical University of Innsbruck, Innsbruck, Austria; Department of Internal Medicine IV – Nephrology and Hypertension, Medical University of Innsbruck, Innsbruck, Austria; Department of Internal Medicine IV – Nephrology and Hypertension, Medical University of Innsbruck, Innsbruck, Austria; INNPATH, Institute of Pathology, Tirol Kliniken Innsbruck, Innsbruck, Austria; Department of Internal Medicine IV – Nephrology and Hypertension, Medical University of Innsbruck, Innsbruck, Austria; Department of Internal Medicine IV – Nephrology and Hypertension, Medical University of Innsbruck, Innsbruck, Austria; Department of Internal Medicine IV – Nephrology and Hypertension, Medical University of Innsbruck, Innsbruck, Austria

**Keywords:** biomarkers, diabetic kidney disease, image-guided biopsy, prevalence, type 2 diabetes

## Abstract

**Background:**

The development of chronic kidney disease (CKD) in about 20%–40% of patients with type 2 diabetes (T2D) aggravates cardiovascular morbidity and mortality. Pathophysiology is of increasing relevance for individual management and prognosis, though it is largely unknown among T2D patients with CKD as histologic work-up is not routinely performed upon typical clinical presentation. However, as clinical parameters do not appropriately reflect underlying kidney pathology, reluctance regarding timely histologic assessment in T2D patients with CKD should be critically questioned. As the etiology of CKD in T2D is heterogeneous, we aim to assess the prevalence and clinical disease course of typical diabetic vs atypical/non-specific vs non-diabetic vs coexisting kidney pathologies among T2D patients with mild-to-moderate kidney impairment [KDIGO stage G3a/A1–3 or G2/A2–3; i.e. estimated glomerular filtration rate (eGFR) 59–45 mL/min irrespective of albuminuria or eGFR 89–60 mL/min and albuminuria >30 mg/g creatinine].

**Methods:**

The Innsbruck Diabetic Kidney Disease Cohort (IDKDC) study aims to enroll at least 65 T2D patients with mild-to-moderate kidney impairment to undergo a diagnostic kidney biopsy. Six-monthly clinical follow-ups for up to 5 years will provide clinical and laboratory data to assess cardio-renal outcomes. Blood, urine and kidney tissue specimen will be biobanked to identify diagnostic and prognostic biomarkers.

**Conclusions:**

While current risk assessment is primarily based on clinical parameters, our study will provide the scientific background for a potential change of the diagnostic standard towards routine kidney biopsy and clarify its role for individual risk prediction regarding cardio-renal outcome in T2D patients with mild-to-moderate kidney impairment.

KEY LEARNING POINTS **What was known:**

- Type 2 diabetes (T2D) is a common disease associated with adverse cardio-renal outcome.
- While 20%–40% of T2D patients develop chronic kidney disease (CKD), the underlying renal pathophysiology is not appropriately reflected by clinical parameters and is largely unknown due to a lack of routine histologic assessment in this population.
- Current national and international guidelines strongly recommend early histologic work-up in patients with CKD of unknown etiology.

**This study adds:**

- This prospective cohort study aims to assess the prevalence and disease course of underlying kidney pathologies in T2D patients with mild-to-moderate CKD as well as to clarify the role of renal biopsy for individual risk prediction regarding cardio-renal outcome in this population.
- The establishment of a longitudinal biobank including kidney tissue, blood and urine samples will help to identify prognostic and therapeutic biomarkers.

**Potential impact:**

- This study could provide the scientific background for a potential change of the diagnostic standard towards routine kidney biopsy in T2D patients with CKD.
- The study will enable precision medicine approaches regarding patient-level predictions of prognosis and treatment response.

## INTRODUCTION

Type 2 diabetes (T2D) affects more than 460 million adults worldwide and more than 30 million in the European Union. This pandemic comes with substantial disease-related morbidity and mortality, as well as high socioeconomic burden [1–3]. The number of T2D-related deaths is more than 3.4 million globally and is expected to double between 2005 and 2030 [4, 5]. In 2021, the global health expenditure for T2D was €167 billion [6]. T2D is a systemic disease associated with multiple macro- and microvascular complications such as myocardial infarction, stroke, retinopathy and chronic kidney disease (CKD) [1, 7, 8]. During disease progression about 20%–40% of T2D patients develop CKD, characterized by an estimated glomerular filtration rate (eGFR) <60 mL/min/1.73 m^2^ and/or albuminuria >30 mg/day [9–11]. Concomitant retinopathy, T2D duration >5 years as well as continuously increasing proteinuria and/or decreasing eGFR over time prompt the clinical diagnosis of “diabetic nephropathy,” as the renal phenotype is considered a consequence of the metabolic disorder. Currently, histologic confirmation is not routinely performed and limited to T2D patients with atypical clinical presentation (e.g. nephrotic syndrome, hematuria or rapid eGFR decline) that points towards an alternative pathogenesis [12, 13]. Classic diabetic nephropathy, however, can still be verified by kidney biopsy in about a third of atypically presenting patients, while the other two-thirds present with either ambiguous histologic lesions or a distinct alternative diagnosis (e.g. focal segmental glomerular sclerosis, membranous or immunoglobulin A nephropathy). In the latter, accurate histologic diagnosis can provide better prognostic potential and might allow causal therapy [14, 15]. In contrast, there is also mounting evidence that only a minority of T2D patients with typical clinical presentation suggestive for diabetic nephropathy exhibit typical diabetic lesions (e.g. glomerular basement membrane thickening, mesangial matrix expansion, nodular glomerulosclerosis and arteriolar pathologies) on kidney tissue level. In this regard, Fioretto *et al*. demonstrated in microalbuminuric T2D patients with preserved eGFR that two-thirds had

either normal or near normal renal structure or atypical/non-specific patterns of kidney injury (i.e. tubulo-interstitial damage or arteriolar hyalinosis without glomerular lesions) after kidney pathology work-up [16]. As none of these patients had any definable non-diabetic kidney disease (e.g. primary glomerulonephritis, genetic kidney diseases, etc.) these histologic findings might reflect the heterogenous pathophysiology of T2D-mediated CKD and/or the presence of concomitant non-specific hypertensive or ischemic kidney injury. The substantial pathophysiologic heterogeneity in T2D patients with CKD is also reflected by the fact that about 50% of T2D patients with reduced eGFR do not present with albuminuria [17–19]. To date, thus, “diabetic nephropathy” has become a solely histologic diagnosis (i.e. presence of typical glomerular, tubulo-interstitial and arteriolar changes in parallel) while kidney dysfunction in T2D patients with typical clinical presentation is referred to as diabetic kidney disease (DKD) in the absence of kidney biopsy work-up. Additionally, specific non-diabetic kidney diseases (e.g. primary glomerulonephritis, minimal change disease, primary or secondary focal segmental glomerulosclerosis, paraprotein-related kidney injury, etc.) that might not be suspected on the basis of clinical signs or urinalysis can be present either alone or in combination with diabetic kidney lesions in T2D patients with CKD [20, 21]. Current national and international guidelines strongly recommend early histologic work-up in patients with CKD of unknown etiology [14]. While this recommendation is consistently applied to the non-diabetic population around the world, kidney biopsy has not been implemented as diagnostic standard in the T2D population despite its potential impact for personalized management [22].

## WORKING HYPOTHESES

We hypothesize that a timely histological assessment of underlying kidney pathologies (diabetic vs non-diabetic vs atypical vs coexisting) among T2D patients with early-stage CKD is of relevance for individual prognosis and patient management based on the following: the exclusive use of clinical parameters (eGFR, albuminuria) for DKD diagnosis may be sufficient to predict prognosis on a cohort level but needs modification to be useful for individual patient care. CKD in T2D is not a uniform disease and clinical parameters do not appropriately reflect the underlying kidney pathology [13, 23, 24]. Thus, histologic work-up and the establishment of a longitudinal biobank including kidney tissue, blood and urine specimen among T2D patients with early-stage CKD will allow better disease phenotyping and help to identify biomarkers for individual disease etiology, course (prognostic biomarkers) and therapeutic response (predictive biomarkers). In this regard, previous systems-biology approaches already identified predictive biomarkers and novel patho-physiologically relevant pathways based on multi-omics data in diabetic CKD patients [25, 26]. Furthermore, diagnosis of non-diabetic CKD such as primary glomerulonephritis, interstitial nephritis, systemic inflammatory or rheumatic diseases with renal involvement is of imminent relevance for therapy and prognosis. However, their prevalence among T2D patients with typical clinical presentation is unknown. Thus, the reluctance regarding routine histologic work-up of CKD pathogenesis in T2D patients has to be critically questioned.

## STUDY AIMS

The main study aim is (i) the histologic assessment of the prevalence and disease trajectory of typical diabetic vs atypical/non-specific vs non-diabetic vs coexisting kidney pathologies among T2D patients with mild-to-moderate impairment of kidney function [Kidney Disease: Improving Global Outcomes (KDIGO) stage G3a/A1–3 or G2/A2–3].

Further aims include: (ii) the assessment of frequency and type of kidney biopsy-related complications; (iii) renal and cardiovascular prognosis and its correlation to the histologic diagnosis; and (iv) establishment of a longitudinal biobank including kidney tissue, blood and urine specimen in order to identify novel predictive biomarkers for CKD disease etiology, course and therapeutic response.

## METHODS

### Overall study design

The IDKDC study is a prospective, longitudinal, single-center, single group, exploratory cohort study designed to evaluate the prevalence and clinical course of typical diabetic vs atypical/non-specific vs non-diabetic vs coexisting kidney pathologies among T2D patients with CKD KDIGO stage G3a/A1–3 or G2/A2–3.

This Austrian Science Fund (FWF) sponsored study will enroll at least 65 patients with mild-to-moderate kidney impairment to undergo a diagnostic kidney biopsy. A 6-monthly clinical follow-up for at least 24 months, but up to 5 years, will provide clinical and laboratory data to assess cardio-renal outcomes. Blood, urine and kidney tissue specimen will be biobanked to identify diagnostic and prognostic biomarkers.

The study adheres to the recommendations of the SPIRIT (Standard Protocol Items: Recommendations for Interventional Trials) reporting guideline [27] and was approved by the Innsbruck Medical University ethics committee (approval number EK1034/2020). Study Registration Number: 20 220 201-2813 (Clinical Trial Center Innsbruck, https://ctc.tirol-kliniken.at).

### Outcome measures

#### Primary endpoint

Biopsy-confirmed prevalence and disease trajectory of typical diabetic vs atypical/non-specific vs non-diabetic vs coexisting kidney pathologies among T2D patients with mild-to-moderate impairment of kidney function (CKD KDIGO stage G3a/A1–3 or G2/A2–3; i.e. eGFR 60–45 mL/min irrespective of albuminuria or eGFR 89–60 mL/min and albuminuria >30 mg/g creatinine).

#### Secondary endpoints

(i) Frequency and type of kidney biopsy-related complications.
(ii) Cumulative incidence of renal and cardiovascular events (see section “Definition of renal and cardiovascular events”) in T2D patients with biopsy-confirmed typical diabetic vs atypical/non-specific vs non-diabetic vs coexisting kidney pathologies.
(iii) Establishment of a longitudinal biobank including kidney tissue, blood and urine samples in order to identify novel predictive biomarkers for CKD disease etiology, course and therapeutic response.

#### Definition of renal and cardiovascular events

Major renal events are defined as a sustained increase in albuminuria of at least 30% including a transition in albuminuria class, >25% decline of measured GFR or >5 mL/min/year decline of measured GFR (for fast progressors), or progression to doubling of serum creatinine, end-stage renal disease or death. Minor renal events are defined as either eGFR decline >12.5% (i.e. reference change value for eGFR; established to discern true changes in kidney function from random fluctuations [28]).

Major adverse cardiovascular events are defined as non-fatal acute myocardial infarction, non-fatal stroke, cardiac revascularization procedure, hospitalization for heart failure, peripheral vascular disease, defined as symptomatic disease and severe limb ischemia leading to an intervention or vascular amputation and death due to cardiovascular cause.

### Eligibility criteria

Inclusion and exclusion criteria are shown in Table 1.

**Table 1:** Key inclusion and exclusion criteria.

Inclusion criteria	Exclusion criteria
Age >18 years	Age >75 years
Diagnosis of T2D based on American Diabetes Association criteria [29]	Active malignancy
Duration of T2D >5 years	Pregnancy
CKD KDIGO stage G3a/A1–3 or G2/A2–3; i.e. eGFR 60–45 mL/min irrespective of albuminuria or eGFR 89–60 mL/min and albumin-to-creatinine ratio ≥30 mg/g	Severe cardiovascular event within the last 180 days prior to study inclusion (e.g. acute coronary syndrome, stroke, transitory ischemic attack, pulmonary embolism, hospitalization due to congestive heart failure with New York Heart Association stage III/IV)
	Contraindication for withholding antiplatelet and/or anticoagulation therapy (10 days prior to or following biopsy)
	Solitary kidney (functional or anatomic)
	Known bleeding disorder or allergy to local anesthetics
	Inability to achieve a blood pressure <150/90 mmHg
	Presence of any disease likely to affect study participation or safety in a negative way from the principal investigator's perspective

Table 1 shows eligibility criteria. CKD KDIGO stage denotes as chronic kidney disease Kidney Disease Improving Global Outcomes stage. eGFR denotes as estimated glomerular filtration rate. T2D denotes as type 2 diabetes.

### Study population and recruitment

Patient recruitment is conducted at the Medical University Innsbruck, Department of Internal Medicine IV – Nephrology and Hypertension. Screening will be performed during routine clinical visits in our department's outpatient clinic, which is the primary place of referral in Tyrol for general practitioners and specialists in internal medicine taking care of T2D patients with incipient impairment of kidney function. We aim at enrolling T2D patients at least 5 years after diagnosis by American Diabetes Association criteria with mild-to-moderate impairment of kidney function (see inclusion criteria above), a population with 8–22 times higher cardiovascular mortality than those with regular kidney function [29, 30]. Inclusion criteria and exclusion criteria, shown in Table 1, were chosen to avoid systematic exclusion of certain subpopulations (see section “Methods of preventing bias”), prioritize safety and minimize risk for adverse events caused by renal biopsy.

### Interventions

At baseline, enrolled participants undergo diagnostic kidney biopsy during a 24 h in-hospital stay (details are provided in the Supplementary Appendix). The retrieval of two biopsy cores with a maximum of two biopsy attempts is intended. The first core sample will be sent for histopathologic assessment at the local pathology facility. The second core sample—if available—will be snap frozen, embedded in Tissue-Tek^©^ O.C.T. compound and stored for biobanking at –80°C.

The retrieval of two biopsy cores (one core for histologic work-up and biobanking, each) with a maximum of two biopsy attempts is mandated by protocol. If only one core is recovered during two biopsy attempts, this core will be primarily sent for histologic work-up and only surplus material is used for biobanking (“diagnostic first” approach). Furthermore, 24-h ambulatory blood pressure monitoring as well as assessment of GFR by iohexol plasma clearance measurement will be performed at baseline and reassessed once a year during follow-up. Collection of clinical and laboratory data as well as blood and urine specimen sampling (50 and 25 mL per visit, each) for longitudinal biobanking at –80°C will be conducted at baseline and during 6-monthly follow-ups. A list with collected data is provided in the Supplementary Appendix.

**Figure 1:**
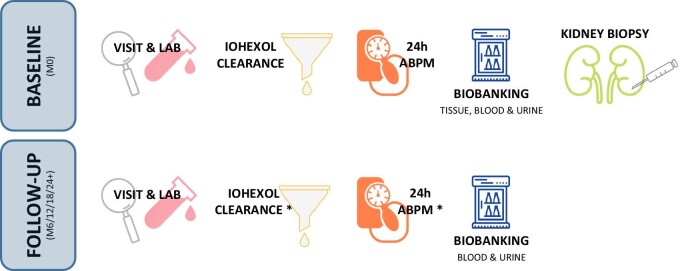
Graphical overview of the IDKDC study. ABPM, ambulatory blood pressure monitoring; M0, Month 0; M6/12/18/24+, 6-monthly follow up for at least 24 months, but up to 5 years. *Iohexol clearance and ABPM is conducted once yearly.

Follow-up will last for at least 24 months but is scheduled for up to 5 years following kidney biopsy, as shown in Fig. 1. Optimization of glucose-lowering medication as well as CKD and cardiovascular disease management is permitted in accordance with practice guidelines throughout the study.

### Assessment of biopsy samples

Histopathologic assessment of kidney biopsies will be evaluated based on a standardized operating procedure according to international consensus guidelines [24, 31–33]. Hematoxylin and eosin, periodic acid–Schiff, Masson trichrome and periodic acid methenamine silver staining, as well as immunohistochemistry, utilizing antibodies against immunoglobulins, complement factors and light chains, will be analyzed by light microscopy. Electron microscopy will be reported separately for the measurement of glomerular basal membrane thickness, detection and localization of deposits and/or fibrils as well as evaluation of podocyte effacement. In addition to establishing a histopathologic diagnosis, the following light microscopic kidney lesions will be semi-quantitatively scored within the glomerular, tubulointerstitial and vascular compartment according to a previously published algorithm [24]:

Endocapillary glomerular inflammation, cellular and fibrocellular crescents, fibrinoid necrosis, mesangial expansion, segmental and global glomerulosclerosis (glomerular compartment); acute tubular injury, inflammation in non-fibrosed interstitial tissue, inflammation in fibrosed interstitial tissue, interstitial fibrosis and tubular atrophy (tubulointerstitial compartment); arteriolar hyalinosis, arteriolar sclerosis, arterial sclerosis (vascular compartment).

The histopathologic categories will be graded by percentage of renal cortical volume or affected glomeruli: none, ≤10%; mild, 11%–25%; moderate, 26%–50%; and severe, >50%.

To better reflect active vs chronic changes, scoring and subsequent grading of chronic kidney pathologies will be conducted according to a previously published approach [33]: global and segmental glomerulosclerosis, tubular atrophy and interstitial fibrosis will be separately scored from 0 to 3 (0, <10%; 1, 10%–25%; 2, 26%–50%; 3, >50% of affected glomeruli or percentage of renal cortex) and arteriosclerosis from 0 to 1 (0, intimal thickening < thickness of media; 1, intimal thickening ≥ thickness of media). The scores are then added to calculate a total kidney chronicity score to grade the overall severity of the chronic lesions into minimal (0–1), mild (2–4), moderate (5–7) and severe (≥8). According to international consensus guidelines, typical diabetic lesions in the glomerulus will be further subcategorized utilizing both light microscopy and electron microscopic morphometry [31, 32]: Glomerular classification ranges from 1 (mild or nonspecific light microscopic changes and electron microscopy-proven glomerular basement thickening), to 2a (mild mesangial expansion), 2b (severe mesangial expansion), 3 (nodular sclerosis) and 4 (advanced diabetic glomerulosclerosis).

### Safety considerations

With the use of state-of-the-art ultrasound-guided biopsy techniques, the complication rate of kidney biopsy is low. In this regard, an analysis of more than 9400 kidney biopsies demonstrated that the incidence of macrohematuria was 3% and blood transfusions were required in 0.9% of cases only. Severe complications, such as embolization of renal segment arteries, surgical interventions or death, are extremely rare if potential contraindications (i.e. uncontrolled hypertension, bleeding diastasis or incompliance) are adequately considered [34].

Kidney biopsy will be performed during a 24-h in-hospital stay using a standardized routine diagnostic technique according to established local standard operating procedures as outlined in the Supplementary Appendix. Kidney biopsy will be conducted after a 12-h fasting period if blood count, high-sensitivity C-reactive protein and coagulation status are within the normal range and blood typing is completed. Before and after the procedure, systemic blood pressure is maintained below 160/90 mmHg. For safety reasons, a maximum of two attempts will be allowed for the retrieval of two kidney biopsy core samples. After sterile wound dressing, strict bed rest in supine position and compression of the biopsy path for 5 h is mandated by protocol followed by loose bed rest until the following morning. Oral food intake is permitted after first non-macrohematuric void only. In case of pain or macrohematuria, a full blood count as well as an ultrasound examination is immediately obtained and a computed tomography scan will be performed in case of suspected bleeding. In all patients, ultrasound evaluation will be performed on the day following kidney biopsy to detect potential hematomas or fistulas. In the absence of any pathologies, patients will be discharged and advised to avoid heavy lifting (>10 kg) and exercise, as well as contact sports (e.g. boxing), for 2 weeks. If hematomas or fistulas are detected, postprocedural management will be adapted accordingly (see Supplementary Appendix).

Nonetheless, an interim safety analysis will be conducted after the first 10 biopsies and then for every additional 20 biopsies.

### Methods of preventing bias

We aim at enrolling a T2D patient cohort that is representative for real-world outpatient nephrology clinics. We thus selected inclusion and exclusion criteria that do not systematically exclude certain subpopulations and perform a systematic screening of newly referred T2D patients in order to identify each patient meeting the inclusion criteria. Furthermore, in order to prevent bias from differential patient retention during follow-up, we aim at maintaining high follow-up rates (>90%) by embedding the follow-up within regular 6-monthly clinical visits, thereby reducing additional strain for study participants. Additionally, repetitive information about relevant study findings will be provided throughout the entire study to keep patients as motivated as possible. Patients will also be actively contacted in case they do not keep their scheduled appointments during follow-up.

### Statistical considerations

In line with the primary study aim, the cohort's sample size is determined to estimate the proportions of histologic phenotypes. Since reliable population-level data on these proportions are absent, the approach is exploratory. Assuming an 80% prevalence of typical diabetic or atypical/non-specific kidney pathologies, a sample size of 65 patients (accounting for a potential 5% dropout rate) will provide a 95% confidence interval with a margin of error of ±10%.

A thorough cross-sectional analysis will describe patient characteristics (demographics, clinical and laboratory parameters) at inclusion, along with the distribution of diagnosed histologic phenotypes and biopsy complications. This will evaluate data usability and set the basis for further analyses. Continuous variables will be described using measures of location and scale (mean, median, standard deviation, interquartile range). Categorical variables will be presented as absolute and relative frequencies. Visual inspection of distributions (histograms, box plots, bar plots) will additionally identify outliers, assess distributional skewness and guide potential data transformations.

Measures of association between the histologic phenotype and various factors will complete the descriptive cross-sectional multivariate analysis. These factors include cardiovascular disease burden, comorbidities, 24-h ambulatory blood pressure monitoring, iohexol clearance, laboratory, and demographic parameters, medical treatments and potential novel predictive biomarkers. Analyses will include (rank) correlations for continuous variables and measures like phi coefficients from contingency tables for categorical variables, along with selected graphical analyses (scatter plots, box plots). Hypothesized differences or dependencies in observables between histologic phenotypes or groups based on demographics and clinical characteristics will be evaluated using appropriate tests (e.g. *t*-test, Wilcoxon test, chi-square test, Fisher's exact test).

In order to evaluate disease progression from a prognostic point of view, patient trajectories in key kidney parameters and CKD staging will be described longitudinally, grouped by histologic phenotype and relevant covariates. Over the follow-up period, average or median changes of the cohort will be assessed and displayed graphically (e.g. slopes, Sankey diagrams).

Time-to-event analyses of cardiovascular and renal endpoints will begin with robust estimates of incidence rates per person-year stratified by histologic phenotype and outcome-specific risk factors. Cumulative events and survival will be further described with Kaplan–Meier estimates, and log-rank tests will be used to assess differences in risk between phenotypes. Depending on the final number of observations, endpoints and the censoring distribution, hazard regressions (e.g. Cox proportional hazard model) may be used to further infer the longitudinal risk associated with the disease phenotype, adjusted for relevant covariates.

The robustness of the proposed analyses will be evaluated by multiple sensitivity and adequate subgroup analyses.

## DISCUSSION

This prospective cohort study will provide the scientific background for a potential change of the current diagnostic standard towards routine kidney biopsy in the management of T2D patients with CKD. Given the lack of evidence regarding CKD etiology among T2D patients with early disease stages and typical clinical presentation, the study will provide data for the prevalence and prognosis of biopsy-confirmed typical diabetic vs atypical/non-specific vs non-diabetic vs coexisting kidney pathologies in this population. Furthermore, novel predictive biomarkers for disease etiology, course and therapeutic response will be identified. Thus, our proposed study will help to solve a previously unmet clinical need with respect to individualized patient management in T2D patients with CKD [35]. While albuminuria and progressive eGFR loss are hallmarks of CKD, their specificity for the underlying etiology is limited as most kidney diseases typically present with these clinical parameters. National and international guidelines recommend establishing a timely kidney biopsy-based diagnosis in patients with CKD of unknown etiology and an eGFR <60 mL/min and/or pathologic urinary sediment and/or persisting albuminuria (in the absence of contraindications). While this has been consistently recommended for the non-diabetic population, it is not routinely applied to the T2D population [36]. In the light of an entirely unknown rate of non-diabetic kidney disease in T2D patients with CKD and typical disease course as well as the fact that clinical characteristics alone are not sufficient to adequately diagnose the underlying kidney pathology, this reluctant biopsy strategy among T2D patients with CKD can be regarded as increasingly problematic. There are two historic explanations for this inconsistency that leaves the evaluation of CKD pathogenesis at the clinical level in T2D patients. (i) As long as CKD treatment was independent of the underlying pathogenesis and mainly based on optimized metabolic and blood pressure control as well as the inhibition of the renin–angiotensin–aldosterone system—which is used to reduce cardiovascular complications in the CKD population in any case—no further therapeutic consequences resulted from histologic work-up [19, 37]. (ii) In the absence of hematuria, gross proteinuria or rapid eGFR decline, treatment for non-diabetic pathologies was also rather limited due to the substantial side effects of available therapeutic options. In recent years, however, therapeutic options dramatically changed with sodium-glucose cotransporter 2 inhibitors and glucagon-like peptide 1 agonists showing nephroprotective effects independent of their glucose-lowering potential as well as the implementation of rituximab in the treatment of inflammatory kidney diseases, thereby replacing rather toxic compounds, e.g. cyclophosphamide [20, 38–42].

Because kidney biopsy has become a rather safe technique with the use of real-time ultrasound guidance, we propose a prospective cohort study to establish a novel biopsy-based standard for CKD diagnosis in T2D patients in order not to systematically disadvantage this population compared with non-diabetic CKD patients.

T2D patients may benefit from a timely biopsy-based diagnosis of a non-diabetic kidney disease etiology as specific prognosis and treatment is not feasible or severely delayed without diagnostic work-up.

In case of histologically confirmed typical diabetic or atypical/non-specific kidney lesions, an immediate benefit from histologic disease verification cannot be guaranteed. Nevertheless, current knowledge suggests that information about kidney tissue biomarker expression and longitudinal changes of blood and urine parameters might be of prognostic and therapeutic relevance in the near future. Recent advancements regarding novel treatment options for DKD such as several ongoing precision medicine approaches aim at elucidating strategies based on better disease phenotyping: while specific therapies are currently initiated and evaluated for effectiveness based on specific clinical phenotypes (e.g. level of albuminuria), future approaches might focus on the underlying pathomechanisms (i.e. pathogenesis of albuminuria). Better molecular phenotyping of individual DKD based on recent technological progress in the field (e.g. multi-omics, systems biology and data analytics approaches) in combination with innovative and individual treatment options for CKD patients demands a precise histologic work-up of T2D patients with kidney function impairment to allow precision medicine approaches in this population [25, 26, 28, 43, 44].

Potential limitations of this cohort study are the single-center character with a rather small sample size and histologic evaluation by a single nephropathologist only. However, challenges in histologic interpretation will be minimized by utilizing a standardized operating procedure according to international consensus guidelines for histopathologic assessment of kidney biopsies (see section “Assessment of biopsy samples”). Study findings among a European primarily Caucasian study population might not be fully representative for a worldwide early-stage CKD population among T2D patients. To avoid selection bias, however, we carefully chose inclusion and exclusion criteria that do not systematically exclude certain subpopulations and perform a systematic screening of newly referred outpatient T2D patients in order to identify each patient meeting the inclusion criteria (see section “Methods of preventing bias”).

Precision medicine aims at advancing disease classification from a descriptive to a pathomechanistic level and to apply personalized disease management based on individual pathophysiology. Novel insights into different disease phenotypes as well as pathophysiology-dependent effects of modern treatment agents call for an updated CKD diagnostic work-up among the T2D population.

Thus, early histologic diagnosis of T2D patients is of utmost importance both for basic science and coming generations of T2D patients as our study findings will provide the basis of future diagnostic standards in T2D patients with CKD. Furthermore, our study will enable precision medicine efforts allowing patient-level predictions of prognosis and treatment response.

## Supplementary Material

sfae109_Supplemental_File

## Data Availability

After study completion data sets will be made available from the corresponding author upon reasonable request.

## STUDY APPROVAL STATEMENT

This study protocol was reviewed and approved by the Innsbruck Medical University ethics committee, approval number EK1034/2020 on 25 May 2020.

## DATA COLLECTION AND PROTECTION ISSUES

All patient associated information will be managed entirely coded. Data collection will be conducted using a standardized evaluation form (electronic case report form) according to Good Clinical Practice (GCP) and Good Laboratory Practice (GLP). All patient-associated samples, clinical data and published results will integrate full anonymity and be subject to privacy protection according to the current European General Data Protection Regulation. All persons involved in the proposed project are subject to medical confidentiality.
