# Supplementary material for: Rationale and design of the Innsbruck Diabetic Kidney Disease Cohort (IDKDC)—a prospective study investigating etiology and progression of early-stage chronic kidney disease in type 2 diabetes
Source: Clin Kidney J. 2024 Apr 11;17(5):sfae109. doi: 10.1093/ckj/sfae109 (PMC11079669; doi:10.1093/ckj/sfae109)
Supplement: sfae109_Supplemental_File [file sfae109_supplemental_file.docx]

Supplementary Appendix

Supplement to: Plattner C, Sallaberger S, Bohn JP, et al. **Rationale and Design of the Innsbruck Diabetic Kidney Disease Cohort (IDKDC) – A Prospective Study investigating Etiology and Progression of Early-Stage Chronic Kidney Disease in Type 2 Diabetes. (Clinical Kidney Journal)**

This appendix has been provided by the authors to give readers additional information about the work.

# Description of methods and planned interventions

At baseline, enrolled participants will undergo a diagnostic kidney biopsy during a 24 h in-hospital stay. In addition to histopathologic assessement based on current pathology guidelines, kidney tissue will be stored for biobanking at -80 ° Celsius. Furthermore, 24 h ambulatory blood pressure monitoring, non-invasive assessment of GFR (Iohexolclearance), collection of clinical and laboratory data as well as serum- and urine sampling for longitudinal biobanking will be conducted at baseline and routine follow-up visits (6 monthly) for at least 24 months but up to 5 years according to protocol version 1.0, 10.02.2020 (*see details below*):

1. **Data collection**

***Baseline***

*Clinical data*

date of visit *DD/MM/YYYY*

date of birth *DD/MM/YYYY*

gender *m/w/d*

ethnicity *caucasian/afroamerican/hisp/other*

time of diagnosis (T2D) *year/unknown/not applicable*

time of diagnosis (arterial hypertension) *year/unknown/not applicable*

start of T2D treatment *year/unknown/not applicable*

start of hypertension treatment *year/unknown/not applicable*

medical history (preexisting conditions)

diabetic retinopathy *yes/no*

congestive heart failure (NYHA stage III, IV) *yes/no*

coronary heart disease (AP, MI, PTCA, CABG) *yes/no*

PAOD (claudicatio, amputation) *yes/no*

cerebrovascular occlusive disease (stroke, TIA) *yes/no*

smoking status *never/currently/ex*

malignancy *yes/no*

medical family history (preexisting illnessess)

kidney disease *yes/no/unknown*

diabetic kidney disease *yes/no/unknown*

hypertension *yes/no/unknown*

T2D *yes/no/unknown*

cardiovascular disease *yes/no/unknown*

malignancy *yes/no/unknown*

body weight kg

height cm

blood pressure mmHg

current medication generic name / dose

*Laboratory data*

blood glucose *mg/dl*

HbA_1_c *%*

serum creatinine *mg/dl*

eGFR (MDRD) *ml/min/1.73m^2^*

albuminuria *mg/g creatinine*

hsCRP *mg/l*

serum cholesterol (total, LDL, HDL) *mg/dl*

serum triglyzerides *mg/dl*

serum potassium *mmol/l*

hemoglobin *g/dl*

serum albumin *mg/dl*

***Follow up*** *(6 monthly)*

*Clinical data*

date of visit *DD/MM/YYYY*

body weight *kg*

blood pressure *mmHg*

diseases (new onset after study inclusion):

diabetic retinopathy *DD/MM/YYYY*

renal replacement therapy (dialysis, transplantation) *DD/MM/YYYY*

coronary heart disease (AP, MI, PTCA, CABG) *DD/MM/YYYY*

cerebrovascular disease (stroke, TIA) *DD/MM/YYYY*

PAOD *DD/MM/YYYY*

hospitalization due to congestive heart failure *DD/MM/YYYY*

death *DD/MM/YYYY*

cardiovascular death *DD/MM/YYYY*

current medication generic name / dose

*Laboratory data*

blood glucose *mg/dl*

HbA_1_c *%*

serum creatinine *mg/dl*

albuminuria *mg/g creatinine*

eGFR (MDRD) *ml/min*

hsCRP *mg/l*

serum cholesterol (total, LDL, HDL) *mg/dl*

serum triglyzeride *mg/dl*

serum potassium *mmol/l*

hemoglobin *g/dl*

serum albumin *mg/dl*

T2D denotes as type 2 diabetes, NYHA denotes as New York Heart Association, AP denotes as stable angina pectoris, MI denotes as myocardial infarction, PTCA denotes as percutaneous transluminal coronary angioplasty, and CABG denotes as coronary artery bypass graft, TIA denotes as transitory ischemic attack, HbA_1_c denotes as glycated hemoglobin A1c, eGFR denotes as estimated glomerular filtration rate, MDRD denotes as “Modification of Diet in Renal Disease”, hsCRP denotes as high-sensitivity C-reactive protein, LDL denotes as low-density lipoprotein, HDL denotes as high density lipoprotein, PAOD denotes as peripheral arterial occlusive disease.

1. **Diagnostic procedures**

***Baseline***

- history
- blood- and urine analysis
- office blood pressure assessment
- weight assessement
- blood- and urine specimen collection (47,7 and 25 ml, respectively) for longitudinal biobanking
- iohexol-clearance (5 ml venous blood)
- 24 h ambulatory blood pressure monitoring
- Kidney biopsy will be performed as standardized routine diagnostic technique according to established local standard operating procedures (SOPs) as follows:

*Before kidney biopsy:*

1. Kidney biopsy will be performed during a 24 h in-hospital stay after a 12 h fasting periode and assessment of blood count, hsCRP, coagulation status, dip stick urinalysis and blood typing. Kidney biopsy will only be conducted if these parameters are within the normal range.
2. Placement of a peripheral venous access.
3. Blood pressure monitoring, target blood pressure <160/90 mmHG, in case of exceeding this threshold, nitroglycerin spray sublingual or urapidil 12.5 mg intravenously will be administered.

*Kidney biopsy:*

Ultrasound-guided biopsy of the left or right lower kidney pole will be performed in prone position under sterile conditions by a nephrologist as follows:

1. Local anaesthesia using 5-10 ml 2% lidocain.
2. 3 mm skin incision at site of biopsy.
3. max. 2 ultrasound-guided biopsy attempts using a Truecut 16 G biopsy device to retrieve 2 kidney biopsy core samples.
4. The first core sample will be sent to the local pathology facility for histopathologic and electronmicroscopic assessement by an experienced nephropathologist.
5. The second core sample – if available – will be snap frozen, embedded in Tissue-Tek^©^ O.C.T. compound and stored for biobanking at -80 ° Celcius within the Nephrology Laboratory.
6. Sterile wound dressing.

*After kidney biopsy:*

1. Strict bed rest in supine position and compression of biopsy path for 5h. Providing the absence of macrohematuria or flank pain, subsequent loose bed rest until next moring.
2. Oral food intake after first non-macrohematuric urine void only.
3. Blood pressure monitoring in 15 minute intervalls for 6 h, target blood pressure <160/90 mmHg, in case of exceeding this threshold, nitroglycerin-spray sublingual or urapidil 12,5 mg intravenously will be administered.
4. Blood count on day of biopsy in case of pain or macrohematuria.
5. Ultrasound or computer tomography scan in case of hypotension or suspected bleeding.

*Day after kidney biopsy:*

Ultrasound evaluation will be performed at the day following kidney biopsy to detect potential hematomas or fistulas. In case of absence of any pathologies, patients will be discharged and advised to avoid heavy lifting (>10 kg) and exercise as well as contact sports (e.g. boxing) for 2 weeks. In the first week after kidney biopsy, heparin in prophylactic dose and platelet inhibitors should be re-initiated in case of absolute indications, e.g. in case of percutaneous transluminal coronary angioplasty or ST-elevation myocardial infarction <1 year, deep venous thrombosis during last 3-6 month, only. Therapeutic anti-coagulation should be witheld for the first 10 days after kidney biopsy.

In case of detection of hematomas and/or fistulas, postprocedural management will be adapted accordingly (e.g. additional follow-up ultrasound examination, extended periode of anti-coagulative agents and/or sports cessation, prolonged hospital stay, etc.).

Once the histopathologic work-up is completed, patients will be informed about the results. In case of relevant prognostic and/or potential therapeutic findings, an immediate follow-up visit will be scheduled.

***Follow-up*** *(6 monthly)*

- history
- blood- and urine analysis
- office blood pressure assessment
- weight assessement
- blood- and urine specimen collection (47,7 and 25 ml, respectively) for biobanking (6 monthly)
- iohexol-clearance (5 ml venous blood, yearly interval)
- 24h ambulatory blood pressure monitoring (yearly interval)

**Description of blood- and urine specimen collection for longitudinal biobanking**

Blood (47,5 ml) and urine (25 ml) specimen collections will be conducted at the day of scheduled clinical visits after a 12 h fasting period:

|  |  | **baseline visit** | **follow-up visits** |
| --- | --- | --- | --- |
| **Specimen** |  | **ml** | **ml** |
| A | Whole blood | 4 | 4 |
| B | Serum | 20 | 28,5 |
| C | Potassium EDTA plasma | 5 | 5 |
| D | Lithium Heparin Plasma | 5 | 5 |
| E | PAXgene^©^ Blood RNA | 5 | 5 |
| F | PAXgene^©^ Blood DNA | 8,5 | - |
| G | urine | 25 | 25 |

EDTA denotes as ethylenediaminetetraacetic acid, RNA as ribonucleic acid and DNA as deoxyribonucleic acid.
